# A Method for Measuring the Weak Value of Spin for Metastable Atoms

**DOI:** 10.3390/e20080566

**Published:** 2018-07-30

**Authors:** Robert Flack, Vincenzo Monachello, Basil Hiley, Peter Barker

**Affiliations:** Department of Physics and Astronomy, University College, Gower Street, London WC1E 6BT, UK

**Keywords:** weak measurement, transition probability amplitude, atomic metastable states

## Abstract

A method for measuring the weak value of spin for atoms is proposed using a variant of the original Stern–Gerlach apparatus. A full simulation of an experiment for observing the real part of the weak value using the impulsive approximation has been carried out. Our predictions show a displacement of the beam of helium atoms in the metastable $2^{3}S_{1}$ state, $\Delta_{w}$, that is within the resolution of conventional microchannel plate detectors indicating that this type of experiment is feasible. Our analysis also determines the experimental parameters that will give an accurate determination of the weak value of spin. Preliminary experimental results are shown for helium, neon and argon in the $2^{3}S_{1}$ and ${}^{3}P_{2}$ metastable states, respectively.

## 1. Introduction

The notion of a weak value introduced by Aharonov, Albert and Vaidman [1,2] has generated wide interest by, not only providing a new possibility of understanding quantum phenomena, but also by generating new experiments to explore deeper aspects of quantum processes. Although Aharonov et al. [1] specifically applied their ideas to spin, Wiseman [3] and Leavens [4] have shown that when applied to the momentum operator, the weak value of the momentum becomes the local momentum used in the Bohm approach [5]. Flack and Hiley [6] have shown that the weak value of the momentum has a close connection with Schwinger’s notion of a transition amplitude [7], a notion that Feynman [8] used to introduce the concept of a path integral. Thus, these ideas open up new ways of thinking about and exploring many puzzling questions that lie at the heart of quantum physics.

Already, Kocsis et al. [9] have carried out a two-slit experiment using single photons to measure the weak value of the transverse momentum, which they then used to construct a series of momentum flow lines that they interpreted as ‘photon trajectories’. Unfortunately, such an interpretation immediately presents a difficulty in that, whereas particles with non-zero rest mass can be localised in the classical limit producing a classical trajectory [10], photons with zero rest mass have no such limit, calling in to question the meaning of a photon trajectory. In spite of this, Flack and Hiley [11] have shown that the flow lines arise from the new concept of a weak Poynting vector.

In a later paper, Mahler et al. [12] extended the earlier results of Kocsis et al. [9] and demonstrated the existence of non-locality in entangled states in an entirely new way. Unfortunately, in the same paper, they argued that the results can be used to support the Bohm mechanics [5]. However, the Bohm approach is based on the non-relativistic Schrödinger equation and does not apply to the electromagnetic field. A test for the Bohm model in this case requires a generalisation of the Bohm approach to field theory. Indeed, such an extension was first outlined by Bohm [13] himself and later extended by Bohm, Hiley and Kaloyerou [14], Holland [15] and Kaloyerou [16]. It was on this basis that Flack and Hiley [11] showed that by introducing a new notion of the weak value of the Poynting vector, the flow lines could be understood in terms of momentum flow.

To test the original Bohm approach, one must use non-relativistic atoms. This paper is concerned with the development of an experiment to measure such weak values, confining our attention to spin (an attempt to measure weak values of momentum was being carried out by Morley, Edmunds and Barker [17] using argon atoms and will not be discussed further in this paper). As far as we know at the time of writing, the only measurements of weak values of spin have been performed on neutrons [18]. No experiments have used atoms. Not only is this of interest in its own right, but it will enable us to experimentally verify the predictions of the Bohm, Schiller and Tiomno [19,20] model of spin. In this model, the spin vector is well defined in terms of Euler angles, which appear in the expression for the weak value and can therefore be measured. A series of recent results related to this model have been presented by Hiley and Van Reeth [21], who show that the spin does not ‘jump’ immediately into an eigenstate. Instead, the spin vector rotates, taking a finite, but measurable time to reach the eigenstate, as originally shown by Dewdney et al. [22,23,24] and Holland [15]. The paper by Hiley and Van Reeth also shows that it is possible to use the weak value to observe this rotation. Hence, it is important to design an experiment to show whether the spin rotates or ‘jumps’.

**Figure 1:**
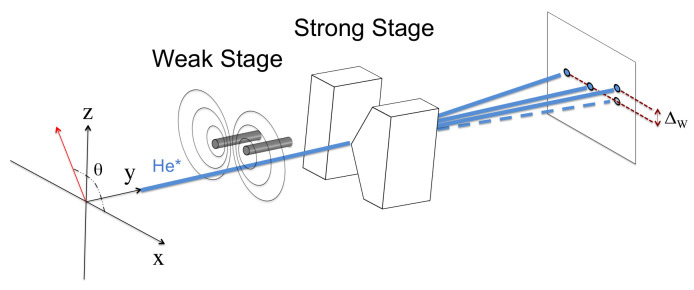
Schematic view of the experimental technique [25]. Helium atoms in the $m_{S}=+1$ metastable state enter from the left, with spin vector angle θ. The atoms pass through the weak and strong S-G magnets before reaching the detector. The displacement due to the weak interaction is $\Delta_{w}$, which is a function of the chosen pre-selected spin state. For simplicity, the $m_{S}=0$ spin state is not shown.

**Figure 2:**
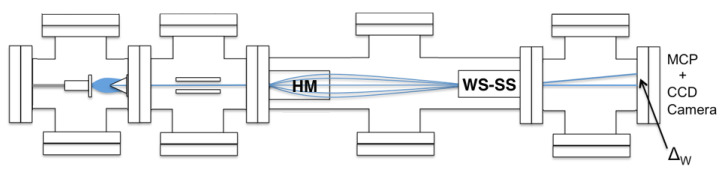
The pulsed helium gas enters from the left. Preparation of the metastable atoms occurs in the first two chambers. In the next chamber, the hexapole magnet (HM) pre-selects the $m_{S}=+1$ state, which moves onto the weak stage (WS), which is comprised of the magnet, and then on to the strong stage (SS) involving the magnet. Finally, the atoms are detected using a micro-channel plate detector (MCP). This figure is reproduced from [25].

The preliminary outline of this experiment was first presented in a conference [25]. For the benefit of the reader, we have reproduced the two key Figure 1 and Figure 2 from this paper. In order to carry out such an experiment, it must be realised that the displacements needed to detect these effects are extremely small. It is therefore important to understand which parameters are critical in limiting the resolution of the changes expected. This paper focuses the discussion on these requirements. To this end, we report on simulations that explore how our apparatus will function. Here, we concentrate on the strong stage (see Figure 2), and to ensure that the apparatus is functioning correctly, we present experimental results involving Stern–Gerlach displacements of various metastable gas species and our ability to efficiently spin select the atomic beam.

## 2. Details of the Experimental Apparatus to Determine Weak Values of Spin

### 2.1. Overview

There are three stages involved in producing the weak value of the spin. Firstly, the atoms are pre-selected in a desired spin state with the spin axis set at a pre-selected angle θ in the *x*-*z* plane; see Figure 1. The atoms then propagate through a weak interaction stage, which, in our case, is comprised of two parallel, current-carrying wires producing an S-G -type field gradient that is very small along the *z*-axis. This stage should not be thought of as constituting a ‘measurement’; it simply introduces a unitary Schrödinger interaction, which produces a small phase change in the wave function carrying information about the weak value.

The final stage involves the actual measurement, using a second conventional S-G magnet, with its strong inhomogeneous magnetic field aligned along the *x*-axis. Note the axes of the weak and strong stage magnets are at right angles to each other. The field of the strong stage magnet must be large enough to clearly separate the spin eigenstates on this axis. It is this separation that enables us to detect the small phase shift, $\Delta_{w}$, induced by the weak stage, as shown in Figure 1. Since the shift $\Delta_{w}$ is small, we must identify and adjust the relevant experimental parameters to maximise the shift. One of the purposes of this paper is to discuss this optimisation.

### 2.2. Stern–Gerlach Simulation Using the Impulse Approximation

The simulation is divided into three parts: the initial conditions, the application of the interaction Hamiltonian in the weak stage using the impulsive approximation [26] and, finally, the action of the strong Stern–Gerlach magnet. This approximation neglects the free evolution of the atoms inside the weak magnet, since this produces negligible effects. The analysis follows the scheme outlined in [27], but in our case, we are using spin-one rather than spin-half particles.

### 2.3. Initial Conditions

Metastable helium atoms in the $2^{3}S_{1}$ state are initially prepared as a pulsed beam and are described by the normalised Gaussian wave packet at time t=0:

(1) $$\psi \left(z,0\right)=\frac{1}{{\left(2\pi \sigma^{2}\right)}^{\frac{1}{4}}}\exp\left(-\frac{z^{2}}{4\sigma^{2}}\right),$$

where σ is the width in position space. The width of the atomic beam is set by passing it through an orifice/skimmer at the entrance of the weak stage. We parametrise the spinor in terms of polar angles θ and ϕ in the following form [28],

(2) $$\xi_{i}\left(\theta ,\phi ,0\right)=\left[\begin{array}{c} \frac{1}{2}\left(1+\sin\left(\theta \right)\right)e^{-i\phi }\\ \frac{1}{\sqrt{2}}\cos\left(\theta \right)\\ \frac{1}{2}\left(1-\sin\left(\theta \right)\right)e^{i\phi } \end{array}\right]=\left[\begin{array}{c} c_{+}\\ c_{0}\\ c_{-} \end{array}\right].$$

The initial orientation of the spin vector angle θ can be seen in Figure 1, where the azimuthal angle ϕ (not shown) is the corresponding angle in the *x*-*y* plane. We set ϕ=0 and only consider variations of the angle θ. Therefore, the initial wave function prior to entering the weak stage is:

(3) $$\Psi_{i}\left(z,0\right)=\psi \left(z,0\right)\xi_{i}\left(\theta \right).$$

### 2.4. Theory of the Weak Stage Process

The atoms then traverse the weak stage magnet, where the wave function evolves under the interaction Hamiltonian, weakly coupling the spin to the centre-of-mass wave function. The interaction Hamiltonian is given by:

(4) $$H_{I}=\mu \left(\hat{s}.B\right),$$

where μ is the magnetic moment, $\hat{s}$ is the spin vector and B the magnetic field. If Δt is the time that the atom spends in the weak field, the wave function as it leaves the weak stage is:

(5) $$\Psi_{f}\left(z,\Delta t\right)=\xi_{f}^{†}\exp\left(-i\frac{\mu \Delta t\frac{\partial B}{\partial z}z{\hat{s}}_{z}}{\hbar }\right)\psi \left(z,0\right)\xi_{i}\left(\theta \right)$$

where we have used the dominant term in the interaction Hamiltonian $B_{z}=\frac{\partial B}{\partial z}z$ [29].

### 2.5. Extracting the Weak Value of Spin

The exponential (phase shift) in Equation (5) can be Taylor expanded:

(6) $$\Psi_{f}\left(z,\Delta t\right)=\langle S_{f}|\left[1-i\frac{\mu \Delta t\frac{\partial B}{\partial z}z{\hat{s}}_{z}}{\hbar }-\frac{1}{2}{\left(\frac{\mu \Delta t\frac{\partial B}{\partial z}z{\hat{s}}_{z}}{\hbar }\right)}^{2}+...\right]|S_{i}\rangle \psi \left(z,0\right),$$

where for convenience, we have written $|S_{i}\rangle$ for $\xi_{i}$ and $\langle S_{f}|$ for $\xi_{f}^{†}$. Hence:

(7) $$\Psi_{f}\left(z,\Delta t\right)=\left[\langle S_{f}|S_{i}\rangle -i\frac{\mu \Delta t\frac{\partial B}{\partial z}z}{\hbar }\langle S_{f}|{\hat{s}}_{z}|S_{i}\rangle -\frac{1}{2}{\left(\frac{\mu \Delta t\frac{\partial B}{\partial z}z}{\hbar }\right)}^{2}\langle S_{f}|{\hat{s}}_{z}^{2}|S_{i}\rangle +\ldots \right]\psi \left(z,0\right).$$

In order to neglect higher order terms in Equation (7), the following inequalities must hold for n≥2 [27,29],

(8) $$\left|{\left(\frac{\mu \Delta t\frac{\partial B}{\partial z}z}{\hbar }\right)}^{n}\langle S_{f}|{\hat{s}}_{z}^{n}|S_{i}\rangle \right|<<\left|\langle S_{f}|S_{i}\rangle \right|$$

and:

(9) $$\left|{\left(\frac{\mu \Delta t\frac{\partial B}{\partial z}z}{\hbar }\right)}^{n}\langle S_{f}|{\hat{s}}_{z}^{n}|S_{i}\rangle \right|<<\left|\left(\frac{\mu \Delta t\frac{\partial B}{\partial z}z}{\hbar }\right)\langle S_{f}|{\hat{s}}_{z}|S_{i}\rangle \right|.$$

In this case, Equation (7) can be expanded to first order:

(10) $$\Psi_{f}\left(z,\Delta t\right)=\left(\langle S_{f}|S_{i}\rangle -i\frac{\mu \Delta t\frac{\partial B}{\partial z}z}{\hbar }\langle S_{f}|{\hat{s}}_{z}|S_{i}\rangle \right)\psi \left(z,0\right),$$

and the transition probability amplitude $\langle S_{f}|S_{i}\rangle$ can be factored out:

(11) $$\Psi_{f}\left(z,\Delta t\right)=\langle S_{f}|S_{i}\rangle \left(1-i\frac{\mu \Delta t\frac{\partial B}{\partial z}z}{\hbar }\frac{\langle S_{f}|{\hat{s}}_{z}|S_{i}\rangle }{\langle S_{f}|S_{i}\rangle }\right)\psi \left(z,0\right).$$

Note that the weak value of the spin, $W=\frac{\langle S_{f}|{\hat{s}}_{z}|S_{i}\rangle }{\langle S_{f}|S_{i}\rangle }$ is in general a complex number with real and imaginary parts. In this case, we are only considering the real part, $W_{Re}$, which becomes,

(12) $$\Psi_{f}\left(z,\Delta t\right)=\langle S_{f}|S_{i}\rangle \left(1-i\frac{\mu \Delta t\frac{\partial B}{\partial z}z}{\hbar }W_{Re}\right)\psi \left(z,0\right).$$

Using the post-selected state, $\xi_{f}^{†}=\left[1/2,1/\sqrt{2},1/2\right]$, the real part of the weak value becomes,

(13) $$W_{Re}=\tan\left(\frac{\theta }{2}\right).$$

In order to cast Equation (11) into an exponential form, the following inequality must be met,

(14) $$L=\frac{\mu \Delta t\frac{\partial B}{\partial z}z}{\hbar }W_{Re}<<1$$

where L<<1 is a limit to be determined [27,29].

As the spread along the *z*-axis is related experimentally to the width of the atomic beam in question [27], *z* can be replaced by σ; therefore, the inequality becomes,

(15) $$L=\frac{\mu \Delta t\frac{\partial B}{\partial z}\sigma }{\hbar }\tan\left(\frac{\theta }{2}\right)<<1.$$

The final wave function after the Gaussian wave packet has traversed both the weak and strong magnets is,

(16) $$\Psi_{f}\left(z,\Delta t\right)=\langle S_{f}|S_{i}\rangle \exp\left(-i\frac{\mu \Delta t\frac{\partial B}{\partial z}z}{\hbar }\tan\left(\frac{\theta }{2}\right)\right)\psi \left(z,0\right).$$

In this experiment, the real part of the weak value of spin will be measured by setting ϕ=0 and varying the angle θ between zero and π.

### 2.6. Free Evolution of the Gaussian Wave Packet at the Detector

After the strong stage, the problem is treated as the free evolution of a Gaussian wave packet by solving the Pauli equation using well-known methods [26]. The probability density can now be computed, giving the form of the wave function as seen by the detector:

(17) $$\begin{array}{c} |\Psi_{D}{\left(z,t\right)|}^{2}=|\langle S_{f}{\left|S_{i}\rangle \right|}^{2}{\left[2\pi \sigma^{2}\left(1+\frac{\hbar^{2}t^{2}}{4m^{2}\sigma^{4}}\right)\right]}^{-\frac{1}{2}}\exp\left[\frac{-{\left(z+utW_{Re}\right)}^{2}}{2\sigma^{2}\left(1+\frac{\hbar^{2}t^{2}}{4m^{2}\sigma^{4}}\right)}\right], \end{array}$$

where *t* is the time of flight from the exit of the strong magnet to the detector. The mean of the post-selected wave function shifts by the value $\Delta_{w}=\left(utW_{Re}\right)=\left(\frac{\mu }{m}\frac{\partial B}{\partial z}\Delta t\right)t\tan\left(\frac{\theta }{2}\right)$, where *u* is the transverse velocity of the helium atoms. This is in contrast to the standard S-G experiment, where the shift is only ut.

As the pre- and post-selected spin states approach orthogonality, θ tends to π and $\Delta_{w}$ increases, but the transition probability decreases. This reduces the number of post-selected events of interest, leading to the need for longer experimental runs. Again, it is important to understand that this effect only arises when the phase shift acquired at the first stage is sufficiently small; see Equation (15). The centre-of-mass wave function is displaced, but its overall shape is maintained after exiting the weak stage.

### 2.7. The Limit and Its Validity

In the literature, the real part of the weak value is given as $\tan\left(\theta /2\right)$. This functional dependence is for an ideal case when the limit in Equation (15) is equal to, or smaller than, an optimal value, which we will call $L_{o}$. For this experiment, it is crucial to know $L_{o}$ in order to successfully measure the well-known $\tan\left(\theta /2\right)$ dependence. If *L* exceeds $L_{o}$, then this will not give the weak value $\tan\left(\theta /2\right)$ because higher order terms begin to dominate. In our case, $L_{o}$ can be determined by analysing two Gaussian wave packets, one describing the first order approximation given by Equation (17) and the other the exact case when no approximation is used, derived from Equation (5).

**Figure 3:**
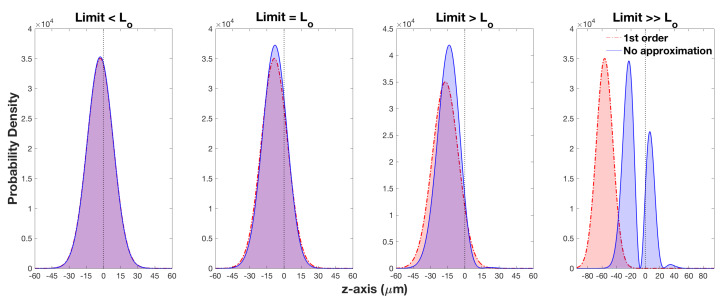
A series of plots showing how the displacement, $\Delta_{w}$, of the Gaussian wave packet is constrained by various limits. The red curve is the first order approximation, which is dominated by tan(θ/2). The blue curve is the exact treatment of the system taking into account all higher order terms. The red and blue curves coincide when the limit $L=L_{o}=0.37$; this is the maximum limit for which the first order approximation holds.

$L_{o}$ is calculated by increasing the inhomogeneous magnetic field in the weak stage only, thus increasing the limit shown in Equation (15); all other variables are held constant. Figure 3 illustrates the behaviour of the two Gaussians. For small values of *L*, the two curves strongly overlap; the point just before the two wave packets deviate is the optimal limit, $L_{o}$. Beyond, $L_{o}$ the first order approximation continues to move to the left, while the full order approximation slowly reverts to that of a standard S-G measurement. Note: this optimal limit is only valid if θ>π/2.

By finding this limit, $L_{o}=0.37$, experimental parameters can be tailored in order to maximise the atomic beam’s displacement due to the weak stage. This is important as certain values of θ produce shifts, which are on the limit of the resolution of our detector. By adjusting experimental parameters in order to meet this limit, displacements for the θ values that would have previously caused an issue can be resolved. As the optimal limit is now fixed, we can rearrange the wave packet deviation $\Delta_{W}$ with respect to this fixed limit.

(18) $$\Delta_{w}=\frac{\mu \frac{\partial B}{\partial z}\left(\Delta t\right)t}{m}\tan\left(\frac{\theta }{2}\right)=\frac{\hbar t}{\sigma m}L_{o}.$$

This shows that the maximum deviation of the wave packet depends on *t* and σ. By changing θ and adjusting other experimental parameters so that $L=L_{o}$, for all values of θ>π/2, we will measure the same displacement, a maximal displacement, and from this, the functional dependence tan(θ/2) can be observed. This is important if we are measuring θ as outlined in Hiley and Van Reeth [21]. Using parameters from our proposed experiment, of which the most important are the atomic velocity of the beam, 1717 m/s, the free flight distance, 2.4 m, the optimal limit, $L_{o}=0.37$, and the width of the beam, σ=0.5μm, our expected displacement, $\Delta_{w}$, is of the order of 20μm.

## 3. Method for the Weak Measurement of Spin for Atomic Systems: Experimental Realisation

### 3.1. Schematic Lay-Out of the Apparatus

A schematic diagram showing the various stages of the measurement is shown in Figure 2. The first step is to produce a beam of metastable helium in the $2^{3}S_{1}$ triplet state. Helium gas at high pressure enters the apparatus from the left and is pulsed into the chamber using an electromagnetic valve, producing a pulsed supersonic beam. The atomic beam is excited using an electron-seeded discharge. Here, the atoms collide with a stream of energetic electrons in a 300 V/cm electric field [30]. The excited gas then passes through a 2 mm-diameter skimmer and travels between two electrically-charged plates to remove the unwanted ionised atoms and free electrons.

The next step is to select a single spin state, in our case the $m_{S}=+1$ state. To do this, we use a hexapole magnet, which focuses this state on to the weak stage magnet (see Figure 2). During this process, the atoms in the $m_{S}=-1$ state are defocused. The $m_{S}=0$, $2^{1}S_{0}$ singlet state and photons are left untouched, but can be removed from the beam by placing a needle across the centre of the magnet. After the beam exits the hexapole magnet, but before it enters the weak stage, it passes through a 50-μm slit; its rotation about the *y*-axis, sets the spin vector angle θ. The pre-selected atomic beam is then passed through a final slit, setting the beam width as required in the limit. The beam width at this point of the process is 0.5μm before entering the weak stage (see Figure 1).

Upon exiting the weak stage, the atomic beam enters the strong stage. Subsequently, the atoms propagate freely onto a detector that consists of two micro-channel plates in a chevron configuration, coupled to a phosphor screen and CCD camera, enabling a resolution of 5μm using centroiding techniques. The measured deflection, $\Delta_{w}$, will be proportional to the weak value of the atomic spin.

### 3.2. Experimental Data Confirming the Correct Functioning of the Last (Post-Selection) Stage

**Figure 4:**
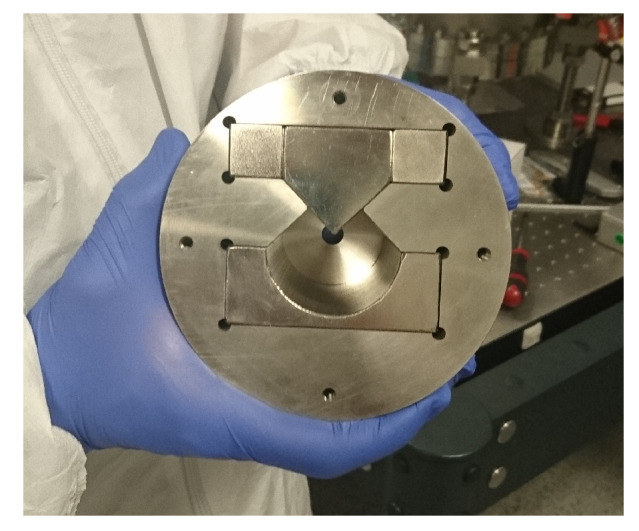
The S-G magnet showing the various grades/shapes of the Nd-Fe-B magnets in the setup in order to achieve a constant field gradient, dB/dx, of 100 T/m.

We check that each stage of the experiment is functioning correctly. Having successfully produced and controlled the metastable helium atoms, we test the functioning of the last stage i.e., the final strong S-G measurement. Here, it is important to ensure that the displacement produced by the strong S-G magnet, for each angular momentum eigenstate, is large enough to be easily resolved. To ensure this, we have used a permanent S-G magnet of length 100 mm. The magnet assembly consists of N38-, N40- and N50-grade Nd-Fe-B magnets, arranged in such a way as to produce a constant field gradient, dB/dx, of 100 T/m over a length of 70 mm (see Figure 4). The force, $F_{x}=-\mu_{x}\;dB/dx$, experienced by an atom in this field is proportional to the magnetic moment of the atom, $\mu_{x}=-g_{J}\mu_{B}m_{J}$, where:

(19) $$g_{J}=\frac{3}{2}+\frac{S\left(S+1\right)-L\left(L+1\right)}{2J\left(J+1\right)}$$

is the Landé g-factor [31].

To carry out this test, we have chosen to the use metastable helium (He*), neon (Ne*) and argon (Ar*). For example, He* has a magnetic moment of $\mu =\pm 2\mu_{B}$, while other noble gases, such as Ne* and Ar*, have magnetic moments of $\mu =\pm 3\mu_{B},\pm \frac{3}{2}\mu_{B}$ and 0, depending on the atoms’, $m_{J}$, state. While He* is in a pure spin state, the other two have a combination of spin and orbital angular momentum.

Experimental S-G distributions for He*, Ne* and Ar* have been measured after first travelling through a collimation region consisting of a 100-μm and 10-μm slit separated by 306.5 mm, producing an atomic beam with an angular divergence of 0.36 mrad. The atoms then travel approximately 2 m before hitting the detector.

**Figure 5:**
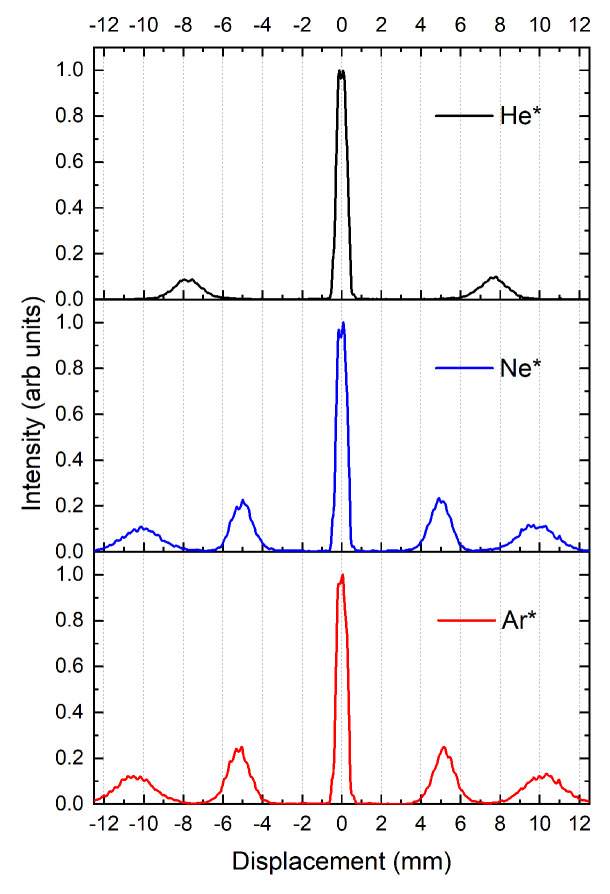
Distribution of three metastable species along the *x*-axis as they travel through a strong S-G magnet and are detected via an MCP detector. From top to bottom, metastable helium (He*) in the $2^{3}S_{1}$ triplet state with $m_{S}=\pm 1,0$, metastable neon (Ne*) and argon (Ar*) in the $3P_{2}$ state with $m_{J}=\pm 2,\pm 1,0$. The states are clearly delineated, indicating that they would be good candidates for measuring weak values of angular momentum. The central peak contribution is larger for all cases due to the double contribution from the m=0 state and photons.

Figure 5 shows the results, confirming that the spin eigenstates for all the gases are sufficiently resolved, giving a displacement of 7.8 mm, for He*, between the $m_{S}=\pm 1$ and $m_{S}=0$ eigenstates, and 10 and 10.4 mm for Ne* and Ar*, respectively, between the $m_{J}=\pm 2$ and $m_{J}=0$ eigenstates. For all systems, the *m* = 0 state, centred at 0 mm, is unaffected by the magnetic field gradient. The observed separations between the states agree with the theoretical predictions, confirming that the strong stage is working correctly.

We have chosen to use metastable helium in the $2^{3}S_{1}$ state as our preferred atom as this gives several advantages:

1. Its magnetic dipole moment, μ, has a magnitude of two Bohr magnetons $\mu =\pm 2\mu_{B}$ [30,32], which allows for sufficient displacement between its three spin eigenstates at the detector.
2. It has a lifetime of approximately 8000 s [33], being unable to decay via electric dipole transitions and the Pauli exclusion principle, i.e., its decay is doubly forbidden. This lifetime is clearly large enough for the atoms to pass through all the stages of the apparatus before decaying. Furthermore, this allows scope for increasing the flight distance with no depreciable effects.
3. Metastable helium atoms have an internal energy of 19.6 eV, the highest of any metastable noble gas species. Upon collision with any surface, it will easily ionise, and the emitted electron is observed with higher efficiency at the microchannel plate (MCP) detector.

All of these characteristics combine to enhance the overall signal strength and sensitivity of the experiment.

### 3.3. The Functioning of the Hexapole Stage

**Figure 6:**
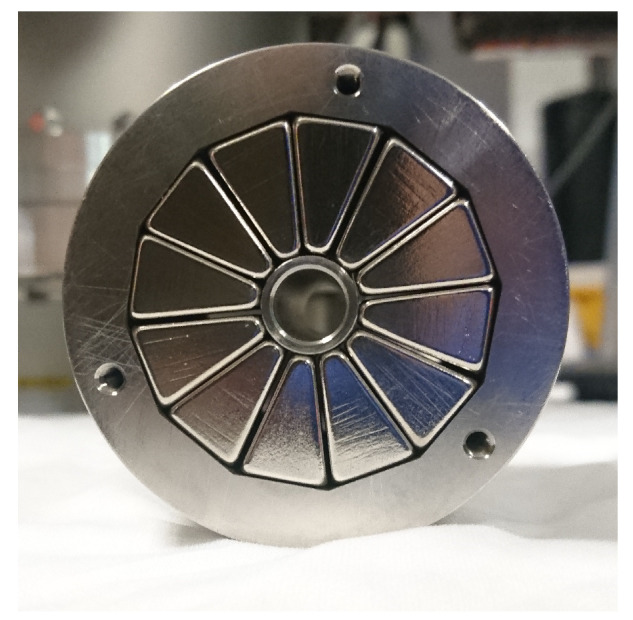
Manufactured hexapole magnet showing the M=12, N42H-grade permanent magnets.

The hexapole magnet contains an array of M=12 segmented nickel-plated N42H-grade permanent magnets, and the array has an ID of 11 mm, an OD of 40 mm and is 60 mm long. The magnetisation direction for each segment is rotated by $120^{\circ }$ with respect to the last. The hexapole magnet is shown in Figure 6, with each individual segment located in a 316LN SShousing.

The magnetic field experienced by an atom in a permanent multipole magnet (produced from *M* segmented pieces) is detailed by Halbach [34] and is shown below:

(20) $$B\left(r\right)=B_{rem}{\left(\frac{r}{r_{1}}\right)}^{n-1}\frac{n}{n-1}\left[1-{\left(\frac{r_{1}}{r_{2}}\right)}^{n-1}\right]\cos^{n}\left(\frac{\epsilon \pi }{M}\right)\frac{\sin\left(\frac{n\epsilon \pi }{M}\right)}{\frac{n\pi }{M}},$$

where $r=\sqrt{x^{2}+z^{2}}$ is the atom’s radial distance from the magnet’s centre. The inner and outer boundaries of the magnet are $r_{1}$ and $r_{2}$, respectively; $B_{rem}$ is the magnetic remanence of the 12 segmented N42H pieces; and for a hexapole magnet, n=3.

The atomic beam is collimated before entering the hexapole magnet by a 5-mm pin hole at its entrance and the 2-mm skimmer, which was located shortly after the supersonic expansion. The two orifices in this collimation region are separated by 440 mm.

**Figure 7:**
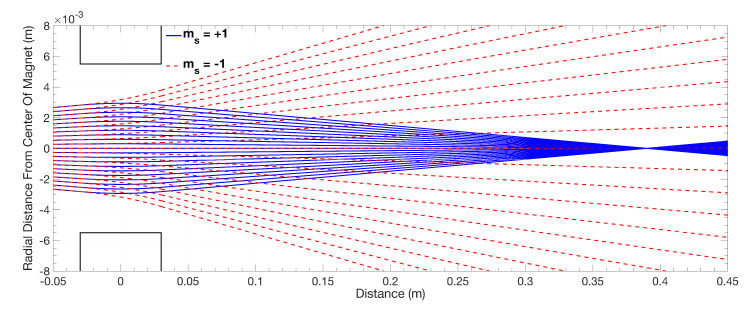
Simulation of a He* beam travelling through the designed hexapole magnet; the dashed red lines signify the $m_{s}=-1$ defocused state, while the blue solid lines signify the $m_{s}=+1$ focused state.

A hexapole magnet utilising these parameters produces a focal point, for He*, which is located approximately 365 mm from the exit of the magnet; see Figure 7. This magnet is also used to reduce the angular divergence of the beam before it passes through our final collimation slit, 1μm, in order to minimise scattering and maximise flux through the slit region.

**Figure 8:**
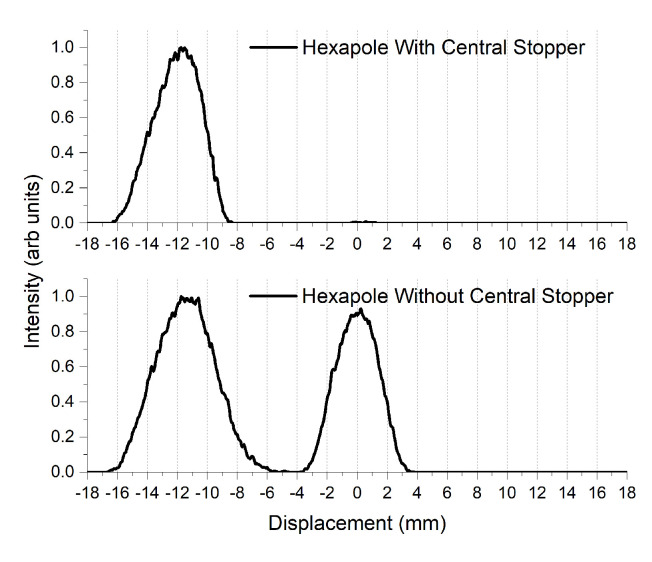
Distribution of the $m_{S}=+1$ and $m_{S}=0$ spin states of the system along the *x*-axis. When a He* beam travels through a permanent hexapole magnet, the $m_{S}=-1$ spin state is defocused and lost to the magnet and the vacuum chamber walls. Note: the width of the atom beam is larger here due to the removal of the collimation region before the S-G magnet for test purposes.

Shortly after leaving this hexapole field, the beam then traverses the strong S-G magnet, producing a well-defined separation of the $m_{S}=+1$ state with complete removal of the $m_{S}=-1$ state, as seen in Figure 8. As can be seen from this figure, the experiment now produces a highly-efficient spin-selected atomic beam, which is required for part of the pre-selection phase of the experiment.

## 4. Conclusions

The experiment described in this paper is designed to measure the real part of the weak value of spin for an atomic system. A full simulation of the process has been carried out giving a prediction of the magnitude of the displacement, $\Delta_{w}$. A limit, $L_{o}$, has been determined defining the range over which the first order approximation holds. Furthermore, we have analysed and optimised the experimental parameters to achieve the largest possible displacement.

We have now been able to sufficiently resolve the spin eigenstates for He* in the *x*-basis, showing that our post-selection region is working as intended. The ability to excite other noble gas species to metastable levels, and sufficiently resolve their angular momentum eigenstates, allows for flexibility in future experiments. Likewise, part of the pre-selection stage is operational, producing a highly spin-selected He* beam with the ability to remove the $m_{S}=0$ and singlet state atoms easily and efficiently from the beam line.

The polarisation mechanics are still to be implemented, allowing us to precisely select the spin vector orientation of the atomic beam, θ. With this, the pre-selection stage is complete. The weak stage S-G magnet has been built and will shortly be introduced into the system. These two extra components complete the main regions of theory and will enable the weak value of spin for He* to be measured.

Using the parameters of our experiment, a shift, $\Delta_{w}$, of the order of 20μm is predicted, which is within our experimental resolution. There is also scope to increase $\Delta_{w}$ by cooling the atomic beam, thus reducing the velocity of the atoms and by reducing the width of the beam before the weak stage. These refinements can increase $\Delta_{w}$ to 20–40 μm. Our experiment is designed to vary the angle θ and thereby show its relationship with $\Delta_{w}$, i.e., tan(θ/2). This means that the weak value can be used to measure the angle θ when it is initially unknown. It is this feature that will enable us to track the change of orientation of the spin vector as outlined in Hiley and Van Reeth [21].
